# Single-Cell Transcriptome Analysis Revealed Heterogeneity and Identified Novel Therapeutic Targets for Breast Cancer Subtypes

**DOI:** 10.3390/cells12081182

**Published:** 2023-04-18

**Authors:** Radhakrishnan Vishnubalaji, Nehad M. Alajez

**Affiliations:** 1 Translational Cancer and Immunity Center (TCIC), Qatar Biomedical Research Institute (QBRI), Hamad Bin Khalifa University (HBKU), Qatar Foundation (QF), Doha P.O. Box 34110, Qatar; 2 College of Health & Life Sciences, Hamad Bin Khalifa University (HBKU), Qatar Foundation (QF), Doha P.O. Box 34110, Qatar

**Keywords:** single cell analysis, breast cancer, molecular subtypes, TNBC, therapeutic targets

## Abstract

Breast cancer (BC) is a heterogeneous disease, which is primarily classified according to hormone receptors and HER2 expression. Despite the many advances in BC diagnosis and management, the identification of novel actionable therapeutic targets expressed by cancerous cells has always been a daunting task due to the large heterogeneity of the disease and the presence of non-cancerous cells (i.e., immune cells and stromal cells) within the tumor microenvironment. In the current study, we employed computational algorithms to decipher the cellular composition of estrogen receptor-positive (ER^+^), HER2^+^, ER^+^HER2^+^, and triple-negative BC (TNBC) subtypes from a total of 49,899 single cells’ publicly available transcriptomic data derived from 26 BC patients. Restricting the analysis to EPCAM^+^Lin^−^ tumor epithelial cells, we identified the enriched gene sets in each BC molecular subtype. Integration of single-cell transcriptomic with CRISPR-Cas9 functional screen data identified 13 potential therapeutic targets for ER^+^, 44 potential therapeutic targets for HER2^+^, and 29 potential therapeutic targets for TNBC. Interestingly, several of the identified therapeutic targets outperformed the current standard of care for each BC subtype. Given the aggressive nature and lack of targeted therapies for TNBC, elevated expression of ENO1, FDPS, CCT6A, TUBB2A, and PGK1 predicted worse relapse-free survival (RFS) in basal BC (*n* = 442), while elevated expression of ENO1, FDPS, CCT6A, and PGK1 was observed in the most aggressive BLIS TNBC subtype. Mechanistically, targeted depletion of ENO1 and FDPS halted TNBC cell proliferation, colony formation, and organoid tumor growth under 3-dimensional conditions and increased cell death, suggesting their potential use as novel therapeutic targets for TNBC. Differential expression and gene set enrichment analysis in TNBC revealed enrichment in the cycle and mitosis functional categories in FDPS^high^, while ENO1^high^ was associated with numerous functional categories, including cell cycle, glycolysis, and ATP metabolic processes. Taken together, our data are the first to unravel the unique gene signatures and to identify novel dependencies and therapeutic vulnerabilities for each BC molecular subtype, thus setting the foundation for the future development of more effective targeted therapies for BC.

## 1. Introduction

Breast cancer (BC) is the most prevalent malignant disease and the second foremost cause of death among women worldwide [1]. BC’s heterogeneity led to its disease classification into hormone receptor-positive, including estrogen receptor-positive (ER^+^) and progesterone receptor-positive (PR^+^); human epidermal growth factor receptor 2 (HER2^+^); and triple-negative BC (TNBC) [2]. This classification led to more targeted therapies for each BC subtype (i.e., tamoxifen for ER^+^ and Herceptin for HER2^+^ BC), which improved the outcome and survival of BC patients [3]. However, the lack of response in some of the patients and disease relapse represent major clinical challenges. While both anti-ER and anti-HER2 were developed to target key genes essential for ER^+^ and HER2^+^ BC, respectively, the development of alternative targeted therapies has been challenging due to the large degree of tumor heterogeneity and the presence of a large number of non-cancerous cells within the tumor, thereby masking key tumor driver genes. In fact, our recent data led to TNBC classification into seven molecular subtypes, several of which were enriched in immune and mesenchymal signatures [4]. Therefore, the identification of unique gene targets essential for each BC molecular subtype is crucial for the development of novel therapeutic interventions to overcome resistance and patients’ relapse. In the current study, we employed single-cell transcriptomic data to delineate the heterogeneity of ER^+^, HER2^+^, and TNBC. Focusing on the Epithelial Cell Adhesion Molecule (EPCAM)^+^ and lineage (Lin)^−^ cancerous cells, we identified the gene sets enriched in each BC subtype, which highlighted inherent cellular heterogeneity, and were able to classify different BC subtypes with high specificity and sensitivity. We subsequently integrated our data with clustered regularly interspaced short palindromic repeats (CRISPR)-Cas9 perturbational gene effects data from the Achilles project [5] and identified the set of genes essential for the survival of ER^+^, HER2^+^, ER^+^HER2^+^, and TNBC. Our data are the first to provide such a comprehensive dependency map for each BC molecular subtype employing single-cell transcriptomic and CRISPR-Cas9 functional screen data. Several of the identified targets predicted a worse prognosis in TNBC, while targeted depletion of ENO1 and FDPS reduced TNBC viability and colony formation and increased cell death. Our data set the framework for future development of novel therapeutic agents to improve the efficacy of existing therapies and to overcome treatment resistance and patients’ relapse.

## 2. Materials and Methods

### 2.1. Single-Cell Data Retrieval and Bioinformatics

Read count matrices from twelve ER^+^, three HER2^+^, two ER^+^HER2^+^, and nine TNBC were retrieved from the GSE176078 dataset [6]. Expression values were first normalized to counts per ten thousand (CPTT) and were then subjected to iterative clustering and guide-gene selection 2 (ICGS2) and uniform manifold approximation and projection (UMAP) dimensionality reduction to identify cell types using a Pearson correlation > 0.3. A detailed description of the computational algorithm employed has been described before [7]. ICGS2 and AltAnalyze pipelines combine multiple complementary subtype detection methods: hierarchical ordered partitioning and collapsing hybrid (HOPACH, sparse non-negative matrix factorization, cluster ‘fitness’, support vector machine) to identify rare and common cell states. ICGS2 reveals cell clusters through a complex process of PageRank downsampling, feature selection ICGS2, dimensionality reduction and clustering (sparse NMF, SNMF), cluster refinement (MarkerFinder algorithm), and subsequent cluster reassignments using support vector machine (SVM). The analysis initially identified the top 500 genes with the highest dispersion, followed by pairwise correlations of variable genes. Dimension reduction with sparse NMF was applied to improve the delineation of cell clusters following HOPACH clustering. The MarkerFinder algorithm was then used to identify rigorously defined cell clusters with unique gene expression for downstream cell cluster assignment, which identified genes positively correlating with an idealized cluster-specific expression profile. Finally, cell cluster assignment was attained from the marker genes identified for sufficiently fitting clusters, based on the cells assigned to the specific SNMF. The AltAnalyze algorithm and source code can be found at (https://github.com/nsalomonis/altanalyze, accessed on 27 March 2023). We initially filtered the expression data for each molecule subtype on EPCAM^+^CD45^−^CD8A^−^CD4^−^CD19^−^CD3E^−^ and then subjected the data to differential expression and MarkerFinder analysis using AltAnalyze v.2.1.3 pipeline.

### 2.2. Discriminant Analyses

To assess the ability of predicted variables (genes identified from the MarkerFinder analysis) to distinguish between ER^+^, HER2^+^, ER^+^HER2^+^, and TNBC, an Orthogonal Projections to Latent Structures Discriminant Analysis (OPLS-DA) classifier was employed for discriminant analyses using Soft Independent Modelling by Class Analogy (SIMCA) software (version 16; Umetrics, Sweden), as we described before [8]. The performance of the model was tested through the use of the Receiver operating characteristic (ROC) curve and calculating the area under the curve (AUC) value. Classification scores were used to assess the sensitivity and specificity of the test by OPLS-DA.

### 2.3. CRISPR-Cas9 Screen Data Retrieval

Genome-wide CRISPR-Cas9 functional screen data were retrieved from the Achilles project [5], and gene effect scores of ≤−0.3 for each BC molecular subtype were included in the analysis.

### 2.4. Protein–Protein Interaction (PPI)

The identified therapeutic candidate genes for each BC molecular subtype were subjected to PPI using the STRING database v 11.5, as described before [9,10]. Defaults settings were used where a minimum required interaction score of 0.4 (medium confidence) was applied. Our analysis using STRING included known interactions (from curated databases or experimentally determined) and predicted interactions (gene neighborhood, gene fusions, and gene co-occurrence), as well as other interactions (textmining, co-expression, and protein homology). The corresponding figure legend illustrates the types of interactions.

### 2.5. Survival Analysis

Survival analysis of the identified potential therapeutic targets was conducted on 442 basal BC patients from the KMplot database [11]. The KMplot database comprises multiple transcriptomic datasets, including breast cancer, with available follow-up and clinical data retrieved from the Gene Expression Omnibus (GEO) repository. Patients were divided into high and low based on median gene expression and were subjected to Kaplan-Meier relapse-free survival (RFS) analysis. The log-rank test was used for curve comparisons, and a log-rank *p* value < 0.05 was considered significant.

### 2.6. RNA-Seq Data Analysis and Bioinformatics

Transcriptomic data from a cohort of 360 TNBC patients were retrieved from the Sequence Read Archive (SRA) database (https://www.ncbi.nlm.nih.gov/sra/SRP157974; accessed on 27 March 2023) using the SRA toolkit v2.9.2 [12]. Paired-end RNA-seq FASTQ files were subsequently pseudo-aligned to the GENCODE release (v33) using KALLISTO 0.4.2.1, as described before [13,14]. Log2 transformed expression data were then used for expression analysis of different TNBC molecule subtypes (Basal-Like Immunosuppressed (BLIS), Immunomodulatory (IM), Luminal Androgen Receptor (LAR), and Mesenchymal (MES)). Detailed characteristics of the 360 TNBC patients are available in the original publication [15]. Differential expression and gene ontology (GO) enrichment analysis were conducted using iDEP.951 (http://bioinformatics.sdstate.edu/idep95/; accessed on 27 March 2023). Briefly, the normalized expression values (TPM, transcript per million) from the 360-patient TNBC cohort were imported into iDEP95.1 and were subjected to log transformation, and low abundant transcripts (<1 TPM) were excluded from the analysis. The cohort was then divided into high and low based on ENO1 and FDPS expression. Differentially expressed genes (1.5 fold-change (fc) and *p* < 0.05 false discovery rate (FDR)-adjusted) were subjected to GO enrichment analysis in iDEP.951, as described before [16].

### 2.7. Cell Culture and Gene Knockdown

Human TNBC (MDA-MB-231 and BT-549 cell lines) were cultured in Dulbecco’s Modified Eagle’s Medium (DMEM) supplemented with 10% fetal bovine serum (FBS) and 1% penicillin/streptomycin (pen-strep); all were purchased from Thermo Scientific (Rockford, IL, USA). Cells were cultured in a humidified incubator at 37 °C under 5% CO_2_. For functional studies, MDA-MB-231 and BT-549 cells (0.168 × 10^6^ cells/mL) were transfected with the selected siRNAs (ENO1 and FDPS) or scrambled negative control. Transfection was performed using a reverse transfection protocol, as we previously described [4]. Briefly, siRNAs (at a 30 nM final concentration) were diluted in 50 μL of Opti-MEM (cat. no. 11058-021; Gibco, Carlsbad, CA, USA), while 1.5 μL of Lipofectamine 2000 (cat. no. 52758; Invitrogen, Carlsbad, CA, USA) was diluted in 50 μL Opti-MEM, and they were subsequently mixed and incubated at ambient temperature for 20 min. One hundred microliters of transfection mixtures were then added to a 12-well tissue culture plate, and subsequently, 300 μL of cells (0.168 × 10^6^ cells/mL) in transfection medium (Opti-MEM) were added to each well. Twenty hours later, the transfection medium (complete DMEM without antibiotics) was added into each well.

### 2.8. Colony Forming Unit (CFU) Assay and Detection of Cell Death Using Fluorescence Microscopy

TNBC cells were first transfected in a 12-well flat-bottom tissue culture plate, as detailed above. On day 5, cells were fixed for 5 min using 4% paraformaldehyde (PFA) and then were washed twice using PBS, followed by staining with crystal violet (0.1% in 10% EtOH) at room temperature for 10 min. The images were then captured and compared to experiment controls. The plates were then allowed to air dry, followed by CFU quantification of dissolved crystal violet in 5% SDS. Absorbance was measured at 590 nm. The experiments were repeated at least twice, and data were presented as the mean ± SD from four replicas.

The Acridine orange/ethidium bromide (AO/EtBr) fluorescence staining method was used to assess apoptosis/necrosis in TNBC cells, as we described before [17]. On day 5 post siRNA transfection, TNBC cells were washed twice with PBS and were then stained with dual fluorescent staining solution (100 μg/mL AO and 100 μg/mL EtBr (AO/EtBr, Sigma Aldrich, St. Louis, MO, USA)) for 2 min. The cells were subsequently observed and imaged under an Olympus IX73 fluorescence microscope (Olympus, Tokyo, Japan). The differential uptake of AO/EtBr allows the identification of viable and non-viable cells. Principally, AO stains nucleated cells with an intact membrane (alive or early apoptosis), while EtBr-positive cells indicated late apoptotic and necrotic cells (cells with a damaged cell membrane), as it normally does not cross the cell membrane of viable cells. For quantitative analysis, the number of dead cells (red) was enumerated in three different fields (10×) using ImageJ (https://imagej.nih.gov/ij/download.html; accessed on 27 March 2023).

### 2.9. Scratch Assay

To assess the migration of ENO1 and FDPS knockdown cells, after transfection on day 5, cells were trypsinized and reseeded in 6 cm cell culture dishes. When they reached confluence, the cell monolayers were scratched using a plastic micropipette tip (yellow tip; 20–200 μL). The cell monolayers were then washed, and the medium was replaced with fresh culture medium. Images of the wounded region were taken immediately (0 h) and after 24 h, using phase-contrast microscopy. Quantification of the wound area was conducted as described before using ImageJ [18].

### 2.10. Organoid Dome Culture

To make 3-dimensional (3D) organoids, cell pellets (250,000 cells/mL) were mixed with overnight-thawed Matrigel (Corning; 356231; Growth Factor Reduced (GFR) Basement Membrane Matrix). Subsequently, multiple drops of cell suspension were plated in pre-warmed (@37 °C) 60 mm Ultra-Low Attachment Culture Dishes (Corning; 3261), then the dishes were placed upside down in a 37 °C, 5% CO_2_ cell culture incubator to allow the droplets to solidify for 20 min, before adding 4–5 mL of expansion medium and further incubation. After one week, organoid formation was observed under the microscope. Quantification of the number of organoids under each treatment condition was conducted using ImageJ software. The number of organoids in three different fields (10×) was enumerated, and the relative number (%) was compared to control siRNA cells.

### 2.11. Statistical Analysis

Statistical analyses for differential expression analyses were conducted in AltAnalyze v.2.1.3. or iDEP.951, using a 1.5 fold change (fc) and FDR-adjusted *p* value < 0.05 as the cutoff, unless stated otherwise. Graphing and pairwise statistical analyses were conducted in GraphPad prism v9.

## 3. Results

### 3.1. Single-Cell Transcriptome Analysis Revealed the Cellular Compositions of Different BC Molecular Subtypes

**Figure 1:**
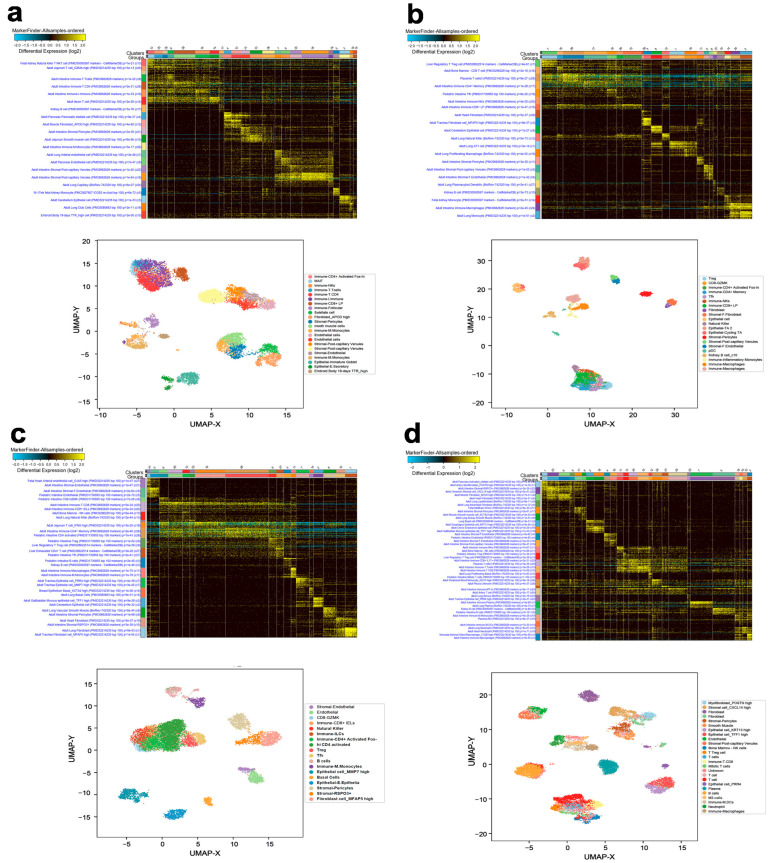
BC heterogeneity delineated by single-cell transcriptome analysis. Single-cell analysis of a total of 16,350 single cells derived from 12 ER^+^ BC (**a**), 7824 single cells derived from 3 HER2^+^ BC (**b**), 11,487 cells derived from 2 ER^+^HER2^+^ BC (**c**), and 14,238 single cells derived from 9 TNBC (**d**) employing AltAnalyze algorithm depicted as heat map. The lower panels represent UMAP dimensionality reduction visualization of the identified cell clusters. The text on the left of each heatmap indicates enriched cell-type markers from the default gene-set enrichment analysis and corresponding “Z” score *p* value.

ICGS2 and UMAP algorithms were employed to delineate the cellular composition of different BC molecular subtypes, employing single-cell transcriptomic data. Clustering patterns and the corresponding UMAP analysis revealed the presence of multiple cell types, including immune cells, fibroblasts, pericytes, and endothelial and epithelial cells, in ER^+^ (Figure 1a and Figure S1). The cellular composition of HER2^+^ highlighted the presence of immune subsets, fibroblasts, stromal, pericytes, and various epithelial cell types (Figure 1b and Figure S2). Tumors from ER^+^HER2^+^ were predominantly infiltrated by various immune subsets and endothelial, stromal, pericyte, and epithelial cells (Figure 1c and Figure S3). TNBC tumors were largely infiltrated by multiple immune cells, myofibroblasts, pericytes, smooth muscle cells, and endothelial, as well as multiple epithelial, subsets (Figure 1d and Figure S4). Our data implied that common gene signatures derived from each BC molecular subtype could potentially be driven by infiltrating microenvironment cells, rather than from cancerous epithelial cells.

### 3.2. Identification of Unique Gene Markers Expressed by Cancerous Epithelial Cells in Each BC Molecular Subtype

**Figure 2:**
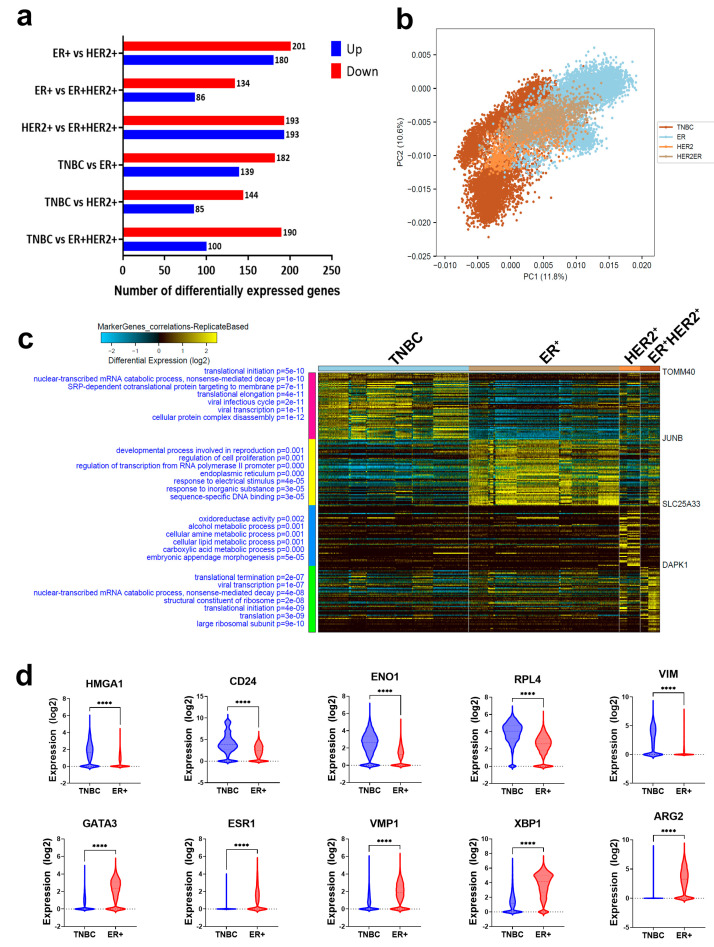
Identification of gene biomarkers enriched in each BC molecular subtype. (**a**) Bar chart depicting the number of differentially expressed genes (DEGs) in ER^+^ vs. HER2^+^, ER^+^ vs. ER^+^HER2^+^, HER2^+^ vs. ER^+^HER2^+^, TNBC vs. ER^+^, TNBC vs. HER2^+^, and TNBC vs. ER^+^HER2^+^. (**b**) PCA illustrating the segregation of each BC molecular subtype based on the identified gene markers. (**c**) Heatmap depicting the enriched gene markers associated with TNBC, ER^+^, HER2^+^, and ER^+^HER2^+^ employing the MarkerFinder algorithm. The text on the left of each heatmap indicates enriched Go functional categories in each BC subtype and the corresponding enrichment *p* values. (**d**) Expression of selected genes enriched in TNBC (upper panel) or ER^+^ (lower panel) based on RNA–Seq data. **** *p* < 0.00005.

**Figure 3:**
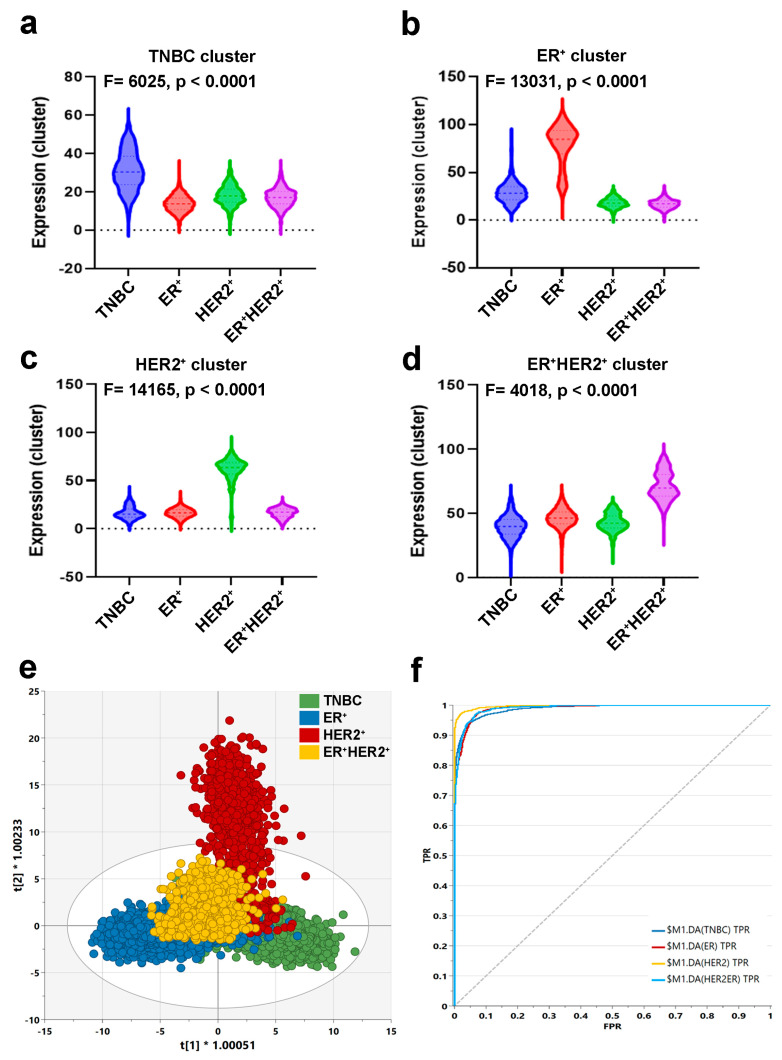
Discriminative analysis based on the identified gene markers. Anova analysis for the identified gene markers enriched in TNBC (**a**), ER^+^ (**b**), HER2^+^ (**c**), and ER^+^HER2^+^ (**d**). Sum score from the identified gene markers for each BC subtype were used for Anova analysis. (**e**) OPLS-DA score plot for the different BC molecular subtypes based on the identified gene markers. (**f**) ROC analysis of the identified gene markers comparing the four BC molecular subtypes.

To identify tumor-derived gene markers expressed by cancerous cells from each BC molecular subtype, we initially filtered the single-cell gene expression data and restricted the analysis to epithelial cells (EPCAM^+^CD45^−^CD8A^−^CD4^−^CD19^−^CD3E^−^). Figure 2a illustrates the differential expression analysis (1.5 FC, FDR-adjusted *p* value < 0.05), which identified 381 differentially expressed genes in ER^+^ vs. HER2^+^ (Table S1), 220 differentially expressed genes in ER^+^ vs. ER^+^HER2^+^ (Table S2), 386 differentially expressed genes in HER2^+^ vs. ER^+^HER2^+^ (Table S3), 321 differentially expressed genes in TNBC vs. ER^+^ (Table S4), 229 differentially expressed genes in TNBC vs. HER2^+^ (Table S5), and 290 differentially expressed genes in TNBC vs. ER^+^HER2^+^ (Table S6). The marker finder algorithm was then used to identify the sets of gene transcripts distinctive for each molecular subtype (TNBC, ER^+^, HER2^+^, and ER^+^HER2^+^), as illustrated in the principal component analysis (PCA) plot (Figure 2b) and the corresponding heatmap (Figure 2c). GO enrichment analysis revealed enrichment in the cellular protein complex disassembly, viral transcription, viral infectious cycle, translational elongation, SRP-dependent cotranslational protein targeting to the membrane, transcribed mRNA catabolic process, nonsense-mediated decay, and translational initiation in TNBC; enrichment in the sequence-specific DNA binding, response to inorganic substances, response to electrical stimulus, endoplasmic reticulum, regulation of transcription from RNA polymerase II promoter, regulation of cell proliferation, and developmental process involved in reproduction in ER^+^; enrichment in the embryonic appendage morphogenesis, carboxylic acid metabolic process, cellular lipid metabolic process, cellular amine metabolic process, alcohol metabolic process, and oxidoreductase activity in HER2^+^; while ER^+^HER2^+^ was most enriched in the large ribosomal subunit, translation, translational initiation, structural constituent of ribosome, transcribed mRNA catabolic process, nonsense-mediated decay, viral transcription, and translational termination (Figure 2c). The list of the top genes enriched in each molecular subtype is provided in Table S7. The expression of selected genes in ER^+^ vs. TNBC is provided in Figure 2d. The sum score for each molecule subtype gene signature demonstrated significant upregulation in the respective TNBC, ER^+^, HER2^+^, and ER^+^HER2^+^ BC molecular subtype (Figure 3a–d). To assess the ability of identified gene markers to discriminate different BC molecular subtypes, we employed OPLS-DA, which revealed remarkable segregation of TNBC, ER^+^, HER2^+^, and ER^+^HER2^+^ based on the identified gene markers (Figure 3e). ROC analysis revealed the excellent performance of the identified gene classifiers in discriminating the four BC molecular subtypes (AUC: TNBC = 0.98, ER^+^ = 0.94, HER2^+^ = 0.99, and ER^+^HER2^+^ = 0.99) (Figure 3f).

### 3.3. Identification of Novel Therapeutic Gene Targets for Each BC Molecular Subtype

**Figure 4:**
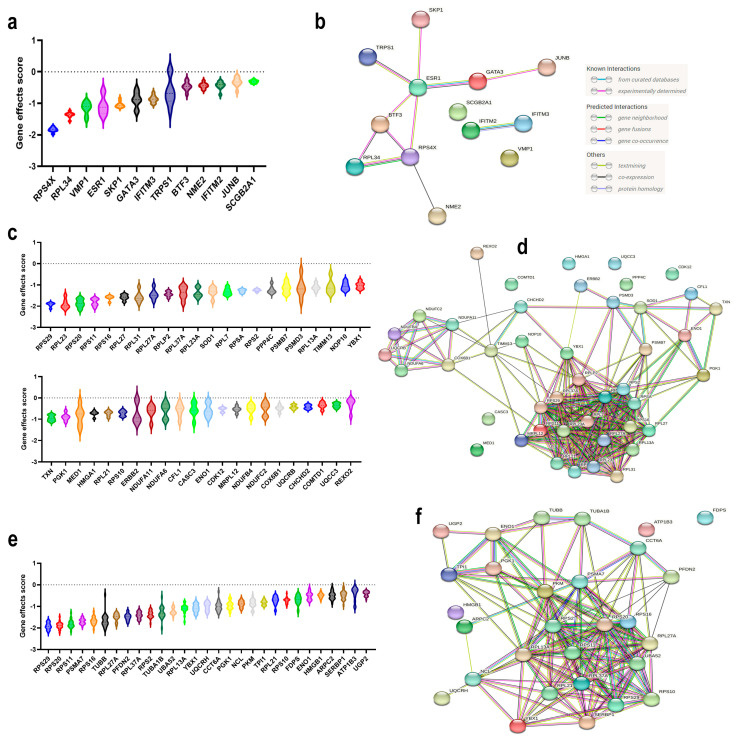
Identification of novel therapeutic targets for each BC molecular subtype employing CRISPR-Cas9 perturbational effects. Violin plots illustrating the perturbational gene effects of the 13 ER^+^ essential genes (**a**), 44 HER2^+^ essential genes (**c**), and 29 TNBC essential genes (**e**). PPI network enrichment analysis for the identified essential genes in (**b**) ER^+^ (number of nodes: 13; number of edges: 11; average node degree: 1.69; avg. local clustering coefficient: 0.623); (**d**) HER2^+^ (number of nodes: 44; number of edges: 225; average node degree: 10.2; avg. local clustering coefficient: 0.625); and (**f**) TNBC (number of nodes: 29; number of edges: 141; average node degree: 9.72; avg. local clustering coefficient: 0.638). CRISPR-Cas9 perturbational gene effects data were retrieved from the Dependency Map database. *Y*-axis represents the perturbational effect scores for the indicated genes.

We subsequently sought to identify potential therapeutic targets for each of the three main BC molecular subtypes. The differentially expressed genes from each BC molecular subtype were crossed with the CRISPR-Cas9 perturbational gene effects data from the Achilles project [5]. Using this strategy, we identified 13 targets in ER^+^ (Figure 4a), 44 targets in HER2^+^ (Figure 4c), and 29 targets in TNBC (Figure 4e).

Interestingly, our data identified RPS4X, RPL34, and VMP1 as having more profound effects than ESR1 for ER^+^ BC, while the majority of the identified therapeutic targets for HER2^+^ BC exhibited more potent inhibitory effects than targeting ERBB2 (i.e., RPS29, etc.). PPI network analysis of the identified ER^+^ gene targets revealed a minimal enrichment score (PPI enrichment *p* value = 0.000313), where the highest GO enrichment was for the response to interferon-alpha (FDR *p* value = 0.0076) (Figure 4b and Figure S5). Stronger enrichment was observed for the gene network identified in HER2^+^ BC (PPI enrichment *p* value ≤ 1.0 × 10^−16^), Figure 4d and Figure S6. The highest enrichment among HER2^+^ gene targets was for the nuclear-transcribed mRNA catabolic process, nonsense-mediated decay, SRP-dependent cotranslational protein targeting to membrane, viral transcription, translational initiation, establishment of protein localization to membrane, and many others (Table S8). Significant enrichments among the TNBC gene targets were observed (PPI enrichment *p* value ≤ 1.0 × 10^−16^) (Figure 4f and Figure S7), where highest enrichment was seen for SRP-dependent cotranslational protein targeting the membrane, viral transcription, nuclear-transcribed mRNA catabolic process, nonsense-mediated decay, translational initiation, and Nucleobase-containing compound catabolic process, in addition to many other enriched GO functional categories (Table S9).

### 3.4. Prognostic Value of the Identified TNBC Therapeutic Targets

**Figure 5:**
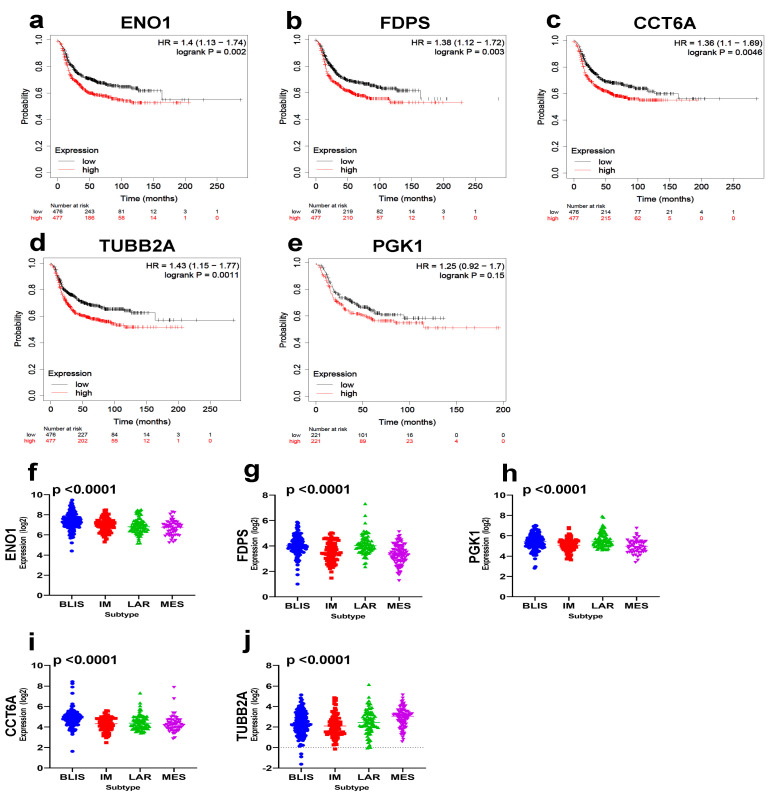
Prognostic significance of the identified TNBC therapeutic targets. Kaplan-Myer RFS analysis for ENO1 (**a**), FDPS (**b**), CCT6A (**c**), TUBB2A (**d**), and PGK1 (**e**) based on median expression in a cohort of 442 basal BCs. Log–rank *p* value is indicated on each plot. Expression of ENO1 (**f**), FDPS (**g**), PGK1 (**h**), CCT6A (**i**), and TUBB2A (**j**) as a function of TNBC molecular subtypes in an independent cohort of TNBC (*n* = 360). Anova *p* value is indicated on each plot.

Given the aggressive nature and lack of effective targeted therapies for TNBC, we focused the remaining part of the study on TNBC. The identified 29 TNBC targets were then subjected to RFS analysis in a large cohort of basal BC (*n* = 442) from the KMplot database [11]. Our data revealed elevated expressions of ENO1, FDPS, CCT6A, TUBB2A, and PGK1 to predict a worse RFS in basal BC (Figure 5a–e).

Interestingly, we observed the highest expression of ENO1 in the aggressive BLIS TNBC subtype, while highest expression of FDPS, PGK1, and CCTA6 was observed in the BLIS and LAR TNBC subtypes (Figure 5f–j). Taken together, our data have highlighted ENO1, FDPS, CCT6A, TUBB2A, and PGK1 as poor prognostic biomarker and potential therapeutic targets for TNBC.

### 3.5. Targeted Depletion of ENO1 and FDPS Reduces TNBC Colony Formation, Viability, Migration, and Growth under 3D Organotypic Culture

**Figure 6:**
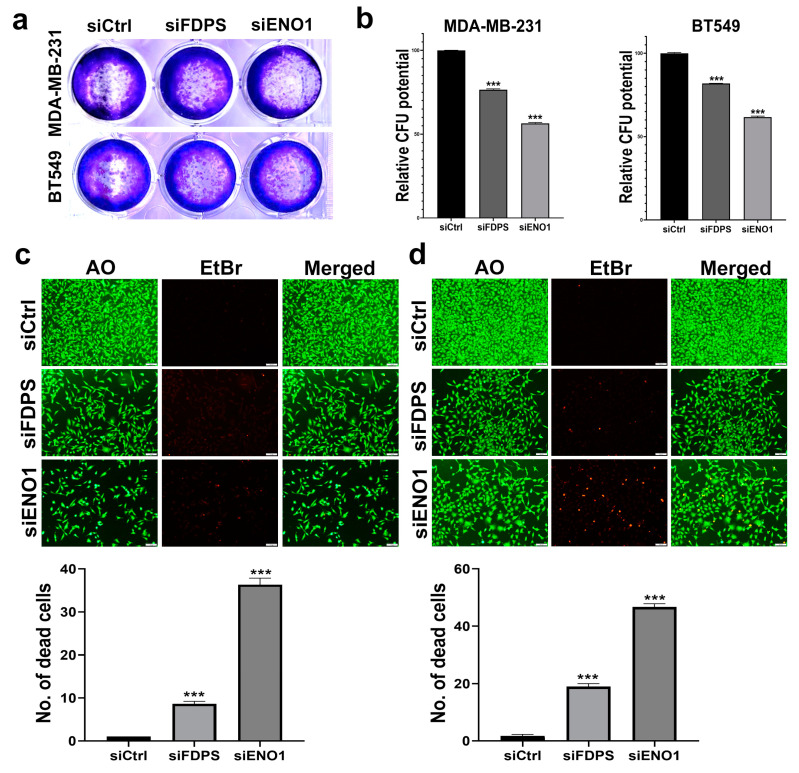
Colony formation and viability assessment of TNBC cells models in response to ENO1 and FDPS depletion. CFU of MDA-MB-231 and BT-549 in response to FDPS and ENO1 depletion (**a**). Quantification of CFU in MDA-MB-231 and BT-549 in response to FDPS and ENO1 depletion (**b**). Data are presented as means ± S.E., *n* = 3. *** *p* < 0.0005. Upper panels show representative live (green) and dead (red) staining of MDA-MB-231 (**c**) and BT-549 (**d**) in response to FDPS and ENO1 depletion. (Scale bar = 1000 μM). Quantification of the number of dead (red) cells under each treatment condition is shown in lower panels. Data are shown as mean number of dead cells in three different fields (10×) ± S.D, *n* = 3. *** *p* < 0.0005.

To provide better understanding of the role of selected therapeutic targets in TNBC, the ramifications of ENO1 and FDPS suppression on TNBC cell viability and growth were assessed using multiple approaches. Those two targets were chosen for their prognostic significance and the limited data currently available in the context of TNBC. Targeted depletion of FDPS1 and ENO1 employing siRNA reduced the colony formation ability of the MDA-MB-231 and BT-549 TNBC models (Figure 6a).

Quantitative analysis of the CFU potential relative to control cells revealed a significant suppression of the colony formation potential of MDA-MB-231 and BT-549, which was more remarkable for ENO1 (56% and 61% CFU, respectively), followed by FDPS (76 and 81% CFU, respectively) (Figure 6b). AO/EtBr staining coupled with fluorescent microscopy revealed the suppression of MDA-MB-231 and BT-549 cell growth and enhanced toxicity in response to ENO1 and FDPS depletion (Figure 6c,d). The quantification of the number of dead (red) cells under each treatment condition is shown in the lower panels, which is concordant with the CFU data.

**Figure 7:**
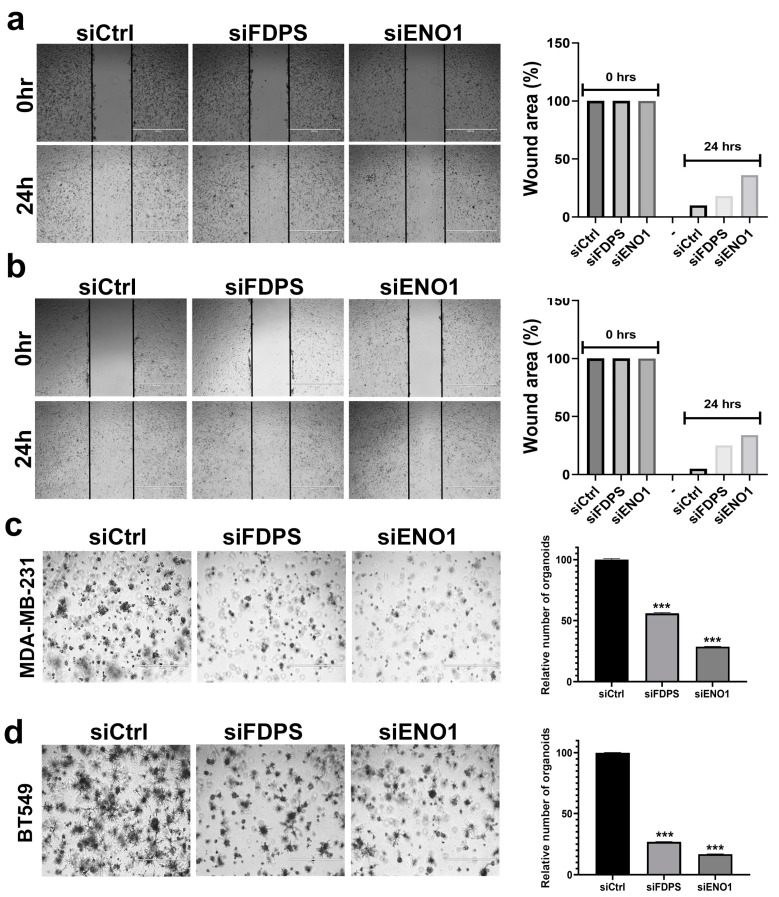
Targeted depletion of FDPS and ENO1 reduces migration and organotypic growth of TNBC cell models. Cell migration (scratch assay) for MDA-MB-231 (**a**) and BT-549 (**b**) TNBC cell models in response to FDPS and ENO1 depletion. Quantification of relative (%) wound area under each treatment condition is shown in the right panels. Inhibition of 3D organoid formation of MDA-MB-231 (**c**) and BT-549 (**d**) TNBC models in response to FDPS and ENO1 depletion. (Scale bar = 1000 μM). Quantification of number of organoids under each treatment condition relative to control is shown in the right panels. Data are shown as mean relative number from three different fields (10×) ± S.D, *n* = 3. *** *p* < 0.0005.

We subsequently assessed the effects of ENO1 and FDPS suppression on the ability of TNBC to migrate and to form colonies under a 3D organotypic culture. Suppression of ENO1, and to a lesser extent FDPS, reduced the migration potential of the MDA-MB-231 and BT-549 cells models (Figure 7a,b). The quantification of the wound area at 24 h, relative to 0 hrs, is shown in the right panels. Concordantly, the suppression of ENO1 and FDPS also inhibited the organotypic growth of TNBC models, thereby corroborating the catastrophic effects of targeting ENO1 and FDPS in TNBC. In agreement with previous data, ENO1 inhibition exhibited more profound effects on organoid formation (Figure 7c,d, right panels).

**Figure 8:**
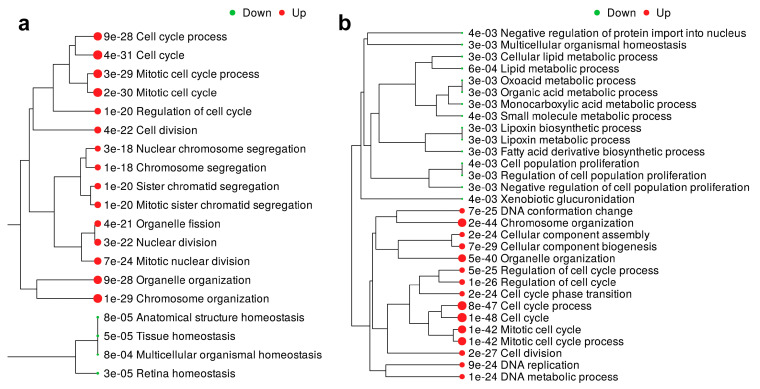
GO enrichment analysis associated with elevated ENO1 and FDPS expression in TNBC. GO enrichment tree in a cohort of 360 TNBC patients divided according to median ENO1 (**a**) and FDPS (**b**) expression. Enrichment *p* value is indicated for each functional category.

To gain better understanding of the potential roles of ENO1 and FDPS in TNBC, a cohort of 360 TNBC patients was divided into high and low, according to the median ENO1 or FDPS expression. Differential expression and GO enrichment analyses revealed a significant association of the elevated ENO1 expression with the biological process associated with the cell cycle, cell division, and organelle organization (Figure 8a and Table S10). Interestingly, GO analysis revealed significant enrichment in several other functional categories in ENO1^high^, including the glycolysis and ATP metabolic processes (Figures S8 and S9). Elevated FDPS expression was associated with the cell cycle, mitosis, organelle organization, and other processes (Figure 8b and Table S11). Those data are in line with our CFU data implicating ENO1 and FDPS in promoting cell proliferation.

## 4. Discussion

BC is a heterogeneous disease where recent advances in the understanding of the disease led to BC’s classification into three major categories: hormone receptor-positive (HR^+^), HER2^+^, and TNBC [19]. Currently, each BC molecular subtype is treated differently; nonetheless, a lack of response and recurrence still represent major clinical challenges [20]. While numerous gene signatures have been devised to better classify different BC subtypes, it is notable that several of those signatures are driven by the presence of a large number of tumor microenvironment cells, rather than by the cancerous cells themselves [21,22,23]. In fact, we recently classified TNBC into seven molecular subtypes; four of those were driven by various immune signatures [4]. Therefore, the identification of the unique tumor biomarkers essential for BC survival could provide more effective targeted therapies, which could be administered in conjunction with existing therapies or as an alternative, as well as to manage patients with disease relapse.

In the current study, we initially delineated the cellular composition of various BC molecular subtypes employing single-cell RNA-Seq data and computational pipelines. Our data revealed the complexity of each BC molecular subtype and identified the many cell types enriched in ER^+^, HER2^+^, ER^+^HER2^+^, and TNBC. Concordant with previous reports, TNBC and HER2^+^ BC were heavily infiltrated by different immune subsets [6,24]. The presence of Neutrophils and plasma cells was predominantly seen in TNBC, while B cells were present in TNBC and HER2^+^HR^+^ tumors. Interestingly, our recent work has highlighted the presence of B cells as a hallmark of TNBC responding to neoadjuvant chemotherapy [25]. The presence of tumor-associated neutrophils was also highest in TNBC, which would be concordant with our findings from the current study [26]. Regulatory T cells (Treg) were enriched in HER2^+^, ER^+^HER2^+^, and TNBC, which would be concordant with published literature correlating Treg infiltration with negative ER and PR status [27]. To identify novel therapeutic targets for each BC molecular subtype, we conducted differential expression analysis focusing only on tumor epithelial cells (EPCAM^+^Lin^−^) and identified the gene set(s) most enriched in each BC molecular subtype. We subsequently crossed our data with CRISPR-Cas9 perturbational functional screen data and identified numerous novel gene targets for each BC molecular subtype. PPI network analysis revealed the association between the identified gene targets based on known interactions (from curated databases or experimentally determined) and predicted interactions (gene neighborhood, gene fusions, and gene co-occurrence), as well as other interactions (textmining, co-expression, and protein homology). Therefore, our PPI analysis was broad and was not restricted to validated interactions, given the relatively small number of identified targets for each BC subtype. The use of community detection algorithms, such as Louvain community detection, could be an added value to the study. Our data identified ESR1 for ER^+^ and ERBB2 for HER2^+^, thus corroborating the effectiveness of our approach. Interestingly, our data identified numerous novel potential therapeutic targets that were more effective than ESR1 and ERBB2 for ER^+^ and HER2^+^ BC, respectively. For instance, our data identified RPS4X, RPL34, and VMP1 as having more inhibitory effects in ER^+^ BC cells compared to ESR1 inhibition. The RPS4X ribosomal protein has been linked to numerous cancer types, including glioblastoma [28], lung adenocarcinoma [29], hepatocellular carcinoma [30], ovarian cancer [31], and many others. RPL34 is another ribosomal protein that has been implicated in several cancer types [32,33,34]. VMP1 is a transmembrane protein that plays an essential role in autophagy [35]. Suppression of VMP1 was shown to inhibit the invasion potential of BC cells [36]. In Glioblastoma, elevated expression of VMP1 was associated with an advanced disease stage, while CRISPR-Cas9-mediated VMP1 depletion inhibited cell proliferation and enhanced cell death [37]. Our analysis identified several novel therapeutic targets for HER2^+^ BC; several of those were components of the ribosomal protein complex. Additionally, we identified numerous non-ribosomal proteins as therapeutic targets for HER2^+^ BC, including SOD1, PPP4C, PSMB7, PSMD3, TIMM13, NOP10, YBX1, TXN, PGK1, MED1, and HMGA1. Several of those identified gene targets have also been implicated in other cancer types [38,39,40].

TNBC represent the most challenging BC subtype due to the lack of targeted therapies and the poor prognosis [41]. In the current study, we identified several potential therapeutic targets that were mainly enriched in TNBC. KEGG pathway analysis of the identified TNBC therapeutic targets revealed ribosome, glycolysis, biosynthesis of amino acids, and carbon metabolism as the main enriched categories. Among the identified targets, TUBB is a beta tubulin protein that acts as a structural component of microtubules, while TUBA1B is also predicted to be involved in microtubule organization. Interestingly, TNBC is oftentimes treated with microtubule inhibitors (i.e., paclitaxel) [42], thus corroborating our data. ENO1, FDPS, PGK1, PKM, PSMA7, NCL, YBX1, SERBP1, UBA52, and TPI1 also constitute promising therapeutic targets for TNBC, based on PPI network analysis. Interestingly, RFS analysis identified ENO1, FDPS, PGK1, TUBB2A, and CCT6A as potential therapeutic targets, which also predicts a worse prognosis. Concordantly, expression of ENO1 was highest in the aggressive BLIS, while the highest expression of FDPS, PGK1, and CCTA6 was observed in the BLIS and LAR TNBC subtypes. Our functional studies also highlighted ENO1 and FDPS as essential TNBC dependencies, thus corroborating their potential utilization as novel therapeutic targets for TNBC. GO enrichment analysis of a TNBC tumor with elevated expression of ENO1 and FDPS revealed the highest enrichment in functional categories associated with the cell cycle and mitosis. Interestingly, we also observed significant enrichment in numerous other functional categories, including canonical glycolysis (Figure S8, *p* = 2.48 × 10^−6^) and ATP metabolic processes (Figure S9, *p* = 1.86 × 10^−6^) in ENO1^high^ TNBC. Our data are in line with previous published reports implicating ENO1 in various aspects of human cancers [43]. Interestingly, we previously reported ENO1 and FDPS, among several other gene targets, as correlating with residual disease in the context of TNBC neoadjuvant chemotherapy resistance, thus corroborating a crucial role for both genes in TNBC [25].

## 5. Conclusions

Our data provide the first dependency map for BC molecular subtypes employing single-cell transcriptomic and functional CRISPR-Cas9 screen data. While several of the identified therapeutic targets in our study are currently in clinical practice, the development of small-molecule inhibitors targeting the additionally identified promising targets from the current study could accelerate the implementation of novel targeted therapies for BC subtypes.

## Data Availability

Accession numbers to all datasets used in the current study are provided in the methods section. Additional data are provided as supplementary data.

## Supplementary Materials

The following supporting information can be downloaded at: https://www.mdpi.com/article/10.3390/cells12081182/s1, Figure S1: Single-cell analysis from twelve ER^+^ BC; Figure S2: Single-cell analysis from three HER2^+^ BC; Figure S3. Single-cell analysis from two ER^+^HER2^+^ BC; Figure S4: Single-cell analysis from nine TNBC; Figure S5: STRING PPI network enrichment analysis for the identified essential genes in ER^+^; Figure S6: STRING PPI network enrichment analysis for the identified essential genes in HER2^+^; Figure S7: STRING PPI network enrichment analysis for the identified essential genes in TNBC; Figure S8: GO enrichment in canonical glycolysis in ENO1^high^ TNBC; Figure S9: GO enrichment in ATP metabolic process in ENO1^high^ TNBC; Table S1: Upregulated genes in ER vs. HER2+ BC; Table S2: Upregulated genes in ER+ vs. ER+HER2+ BC; Table S3: Upregulated genes in HER2+ vs. ER+HER2+ BC; Table S4: Differentially expressed genes in TNBC vs. ER+ BC; Table S5: Differentially expressed genes in TNBC vs. HER2+ BC; Table S6: Differentially expressed genes in TNBC vs. ER+HER2+ BC; Table S7: Marker genes most enriched in the indicated BC molecular subtype; Table S8: PPI enrichment analysis for the 44 therapeutic targets in HER2^+^ BC; Table S9: PPI enrichment analysis for the 29 therapeutic targets in TNBC; Table S10: Differentially expressed genes in ENO1 high vs. low TNBC (1.5 fc, *p* < 0.05 FDR); Table S11: Differentially expressed genes in FDPS high vs. low TNBC (1.5 fc, *p* < 0.05 FDR).

## Abbreviations

BC: Breast cancer; ER, Estrogen receptor; PR, Progesterone receptor; TNBC, Triple-negative BC; HER2, Human epidermal growth factor receptor 2; EPCAM, Epithelial cell adhesion molecule; Lin, lineage; CRISPR, Clustered regularly interspaced short palindromic repeats; CPTT, Counts per ten thousand; ICGS2, Iterative clustering and guide-gene selection 2; UMAP, Uniform manifold approximation and projection; HOPACH, Hierarchical ordered partitioning and collapsing hybrid; SNMF, Sparse non-negative matrix factorization; GEO, Gene expression omnibus; OPLS-DA, Orthogonal projections to latent structures discriminant analysis; SIMCA, Soft Independent Modelling by Class Analogy; PPI, Protein–protein interaction; SRA, Sequence read archive; BLIS, Basal-like immunosuppressed; IM, Immunomodulatory; LAR, Luminal androgen receptor; MES, Mesenchymal; TPM, Transcript per million; GO; Gene ontology; FDR, False discovery rate; DMEM, Dulbecco’s Modified Eagle’s Medium; FBS, Fetal bovine serum; PFA, Paraformaldehyde; AO, Acridine orange; EtBr, Ethidium bromide; ROC, Receiver operating characteristic; AUC, Area under the curve; PCA, Principal component analysis; RFS, Relapse free survival; 3D, 3-Dimensional; CFU, Colony formation unit.

## References

[1] Mattiuzzi C., Lippi G. (2019). Current cancer epidemiology. J. Epidemiol. Glob. Health.

[2] Onitilo A.A., Engel J.M., Greenlee R.T., Mukesh B.N. (2009). Breast cancer subtypes based on ER/PR and Her2 expression: Comparison of clinicopathologic features and survival. Clin. Med. Res..

[3] Lukasiewicz S., Czeczelewski M., Forma A., Baj J., Sitarz R., Stanislawek A. (2021). Breast Cancer-Epidemiology, Risk Factors, Classification, Prognostic Markers, and Current Treatment Strategies-An Updated Review. Cancers.

[4] Elango R., Vishnubalaji R., Shaath H., Alajez N.M. (2021). Molecular subtyping and functional validation of TTK, TPX2, UBE2C, and LRP8 in sensitivity of TNBC to paclitaxel. Mol. Ther. Methods Clin. Dev..

[5] Meyers R.M., Bryan J.G., McFarland J.M., Weir B.A., Sizemore A.E., Xu H., Dharia N.V., Montgomery P.G., Cowley G.S., Pantel S. (2017). Computational correction of copy number effect improves specificity of CRISPR-Cas9 essentiality screens in cancer cells. Nat. Genet..

[6] Wu S.Z., Al-Eryani G., Roden D.L., Junankar S., Harvey K., Andersson A., Thennavan A., Wang C., Torpy J.R., Bartonicek N. (2021). A single-cell and spatially resolved atlas of human breast cancers. Nat. Genet..

[7] Venkatasubramanian M., Chetal K., Schnell D.J., Atluri G., Salomonis N. (2020). Resolving single-cell heterogeneity from hundreds of thousands of cells through sequential hybrid clustering and NMF. Bioinformatics.

[8] Vishnubalaji R., Abdel-Razeq H., Gehani S., Albagha O.M.E., Alajez N.M. (2022). Identification of a Gene Panel Predictive of Triple-Negative Breast Cancer Response to Neoadjuvant Chemotherapy Employing Transcriptomic and Functional Validation. Int. J. Mol. Sci..

[9] Szklarczyk D., Gable A.L., Nastou K.C., Lyon D., Kirsch R., Pyysalo S., Doncheva N.T., Legeay M., Fang T., Bork P. (2021). The STRING database in 2021: Customizable protein-protein networks, and functional characterization of user-uploaded gene/measurement sets. Nucleic Acids Res..

[10] Vishnubalaji R., Elango R., Alajez N.M. (2022). LncRNA-Based Classification of Triple Negative Breast Cancer Revealed Inherent Tumor Heterogeneity and Vulnerabilities. Noncoding RNA.

[11] Lanczky A., Gyorffy B. (2021). Web-Based Survival Analysis Tool Tailored for Medical Research (KMplot): Development and Implementation. J. Med. Internet Res..

[12] Leinonen R., Sugawara H., Shumway M., International Nucleotide Sequence Database Collaboration (2011). The sequence read archive. Nucleic Acids Res..

[13] Bray N.L., Pimentel H., Melsted P., Pachter L. (2016). Near-optimal probabilistic RNA-seq quantification. Nat. Biotechnol..

[14] Shaath H., Elango R., Alajez N.M. (2021). Molecular Classification of Breast Cancer Utilizing Long Non-Coding RNA (lncRNA) Transcriptomes Identifies Novel Diagnostic lncRNA Panel for Triple-Negative Breast Cancer. Cancers.

[15] Jiang Y.Z., Ma D., Suo C., Shi J., Xue M., Hu X., Xiao Y., Yu K.D., Liu Y.R., Yu Y. (2019). Genomic and Transcriptomic Landscape of Triple-Negative Breast Cancers: Subtypes and Treatment Strategies. Cancer Cell.

[16] Ge S.X., Son E.W., Yao R. (2018). iDEP: An integrated web application for differential expression and pathway analysis of RNA-Seq data. BMC Bioinform..

[17] Vishnubalaji R., Elango R., Manikandan M., Siyal A.A., Ali D., Al-Rikabi A., Hamam D., Hamam R., Benabdelkamel H., Masood A. (2020). MicroRNA-3148 acts as molecular switch promoting malignant transformation and adipocytic differentiation of immortalized human bone marrow stromal cells via direct targeting of the SMAD2/TGFbeta pathway. Cell Death Discov..

[18] Wong S.L., Demers M., Martinod K., Gallant M., Wang Y., Goldfine A.B., Kahn C.R., Wagner D.D. (2015). Diabetes primes neutrophils to undergo NETosis, which impairs wound healing. Nat. Med..

[19] Januskeviciene I., Petrikaite V. (2019). Heterogeneity of breast cancer: The importance of interaction between different tumor cell populations. Life Sci..

[20] Riggio A.I., Varley K.E., Welm A.L. (2021). The lingering mysteries of metastatic recurrence in breast cancer. Br. J. Cancer.

[21] Tsang J.Y.S., Tse G.M. (2020). Molecular Classification of Breast Cancer. Adv. Anat. Pathol..

[22] Bareche Y., Buisseret L., Gruosso T., Girard E., Venet D., Dupont F., Desmedt C., Larsimont D., Park M., Rothe F. (2020). Unraveling Triple-Negative Breast Cancer Tumor Microenvironment Heterogeneity: Towards an Optimized Treatment Approach. J. Natl. Cancer Inst..

[23] Wang S., Zhang Q., Yu C., Cao Y., Zuo Y., Yang L. (2021). Immune cell infiltration-based signature for prognosis and immunogenomic analysis in breast cancer. Brief Bioinform..

[24] Stanton S.E., Adams S., Disis M.L. (2016). Variation in the Incidence and Magnitude of Tumor-Infiltrating Lymphocytes in Breast Cancer Subtypes: A Systematic Review. JAMA Oncol..

[25] Vishnubalaji R., Alajez N.M. (2021). Transcriptional landscape associated with TNBC resistance to neoadjuvant chemotherapy revealed by single-cell RNA-seq. Mol. Ther. Oncolytics.

[26] Soto-Perez-de-Celis E., Chavarri-Guerra Y., Leon-Rodriguez E., Gamboa-Dominguez A. (2017). Tumor-Associated Neutrophils in Breast Cancer Subtypes. Asian Pac. J. Cancer Prev..

[27] Liu F., Lang R., Zhao J., Zhang X., Pringle G.A., Fan Y., Yin D., Gu F., Yao Z., Fu L. (2011). CD8^+^ cytotoxic T cell and FOXP_3_^+^ regulatory T cell infiltration in relation to breast cancer survival and molecular subtypes. Breast Cancer Res. Treat..

[28] Li R., Jiang Q., Tang C., Chen L., Kong D., Zou C., Lin Y., Luo J., Zou D. (2022). Identification of Candidate Genes Associated With Prognosis in Glioblastoma. Front. Mol. Neurosci..

[29] Bi G., Zhu D., Bian Y., Huang Y., Zhan C., Yang Y., Wang Q. (2021). Knockdown of GTF2E2 inhibits the growth and progression of lung adenocarcinoma via RPS4X in vitro and in vivo. Cancer Cell Int..

[30] Zhou C., Liu C., Liu W., Chen W., Yin Y., Li C.W., Hsu J.L., Sun J., Zhou Q., Li H. (2020). SLFN11 inhibits hepatocellular carcinoma tumorigenesis and metastasis by targeting RPS4X via mTOR pathway. Theranostics.

[31] Tsofack S.P., Meunier L., Sanchez L., Madore J., Provencher D., Mes-Masson A.M., Lebel M. (2013). Low expression of the X-linked ribosomal protein S4 in human serous epithelial ovarian cancer is associated with a poor prognosis. BMC Cancer.

[32] Du C., Wang T., Jia J., Li J., Xiao Y., Wang J., Mao P., Wang N., Shi L., Wang M. (2022). Suppression of RPL34 Inhibits Tumor Cell Proliferation and Promotes Apoptosis in Glioblastoma. Appl. Biochem. Biotechnol..

[33] Zhu Y., Ren C., Jiang D., Yang L., Chen Y., Li F., Wang B., Zhang Y. (2021). RPL34-AS1-induced RPL34 inhibits cervical cancer cell tumorigenesis via the MDM2-P53 pathway. Cancer Sci..

[34] Liu H., Liang S., Yang X., Ji Z., Zhao W., Ye X., Rui J. (2015). RNAi-mediated RPL34 knockdown suppresses the growth of human gastric cancer cells. Oncol. Rep..

[35] Ropolo A., Grasso D., Pardo R., Sacchetti M.L., Archange C., Lo Re A., Seux M., Nowak J., Gonzalez C.D., Iovanna J.L. (2007). The pancreatitis-induced vacuole membrane protein 1 triggers autophagy in mammalian cells. J. Biol. Chem..

[36] Sauermann M., Sahin O., Sultmann H., Hahne F., Blaszkiewicz S., Majety M., Zatloukal K., Fuzesi L., Poustka A., Wiemann S. (2008). Reduced expression of vacuole membrane protein 1 affects the invasion capacity of tumor cells. Oncogene.

[37] Lin W., Sun Y., Qiu X., Huang Q., Kong L., Lu J.J. (2021). VMP1, a novel prognostic biomarker, contributes to glioma development by regulating autophagy. J. Neuroinflamm..

[38] Glasauer A., Sena L.A., Diebold L.P., Mazar A.P., Chandel N.S. (2014). Targeting SOD1 reduces experimental non-small-cell lung cancer. J. Clin. Investig..

[39] Yang Y., Leonard M., Luo Z., Yeo S., Bick G., Hao M., Cai C., Charif M., Wang J., Guan J.L. (2021). Functional cooperation between co-amplified genes promotes aggressive phenotypes of HER2-positive breast cancer. Cell Rep..

[40] Zanin R., Pegoraro S., Ros G., Ciani Y., Piazza S., Bossi F., Bulla R., Zennaro C., Tonon F., Lazarevic D. (2019). HMGA1 promotes breast cancer angiogenesis supporting the stability, nuclear localization and transcriptional activity of FOXM1. J. Exp. Clin. Cancer Res..

[41] Bianchini G., Balko J.M., Mayer I.A., Sanders M.E., Gianni L. (2016). Triple-negative breast cancer: Challenges and opportunities of a heterogeneous disease. Nat. Rev. Clin. Oncol..

[42] Schmid P., Adams S., Rugo H.S., Schneeweiss A., Barrios C.H., Iwata H., Dieras V., Hegg R., Im S.A., Shaw Wright G. (2018). Atezolizumab and Nab-Paclitaxel in Advanced Triple-Negative Breast Cancer. N. Engl. J. Med..

[43] Huang C.K., Sun Y., Lv L., Ping Y. (2022). ENO1 and Cancer. Mol. Ther. Oncolytics.

