# Supplementary figures and images for: Single-Cell Transcriptome Analysis Revealed Heterogeneity and Identified Novel Therapeutic Targets for Breast Cancer Subtypes
Source: Cells. 2023 Apr 18;12(8):1182. doi: 10.3390/cells12081182 (PMC10137100; doi:10.3390/cells12081182)

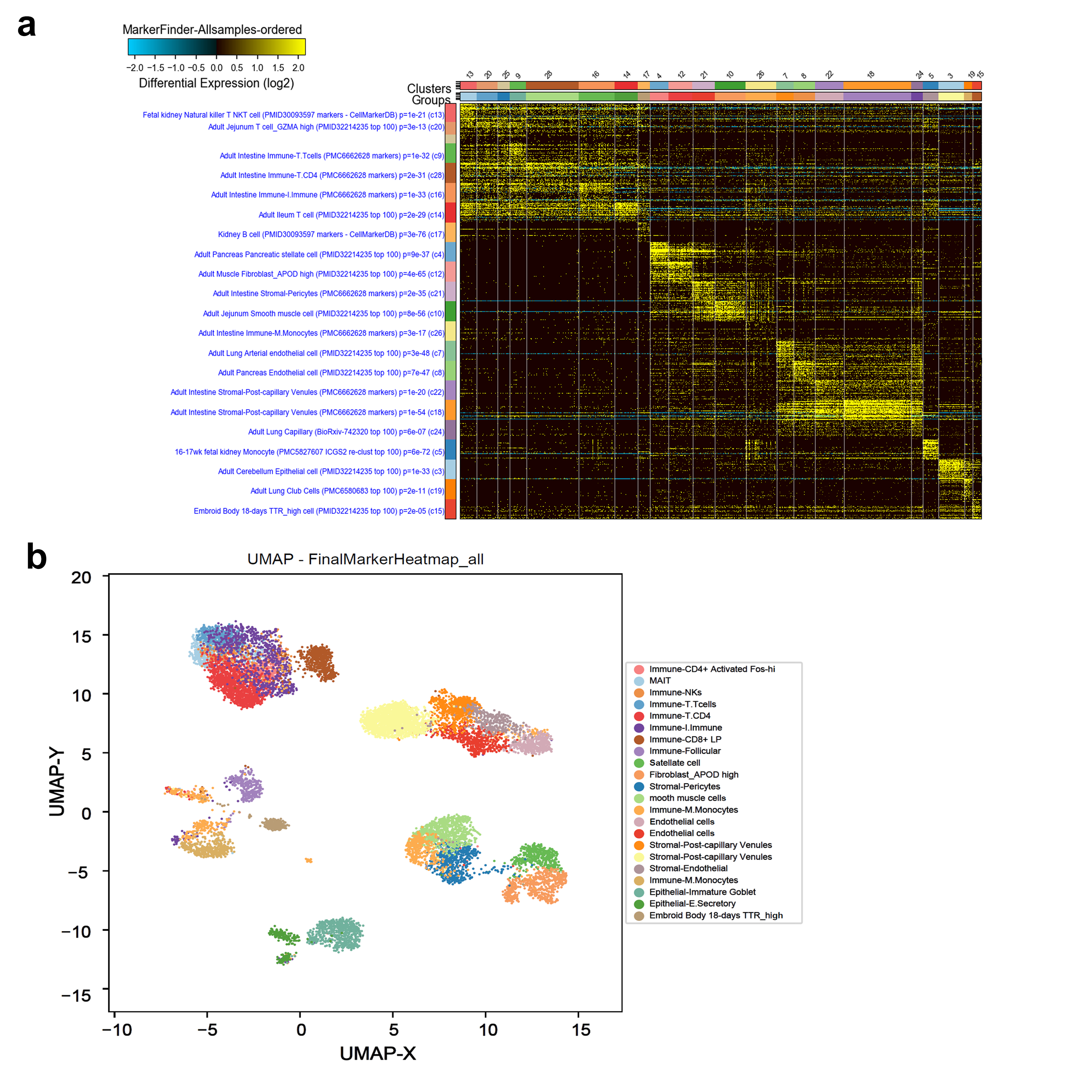

Supplement: Supplementary file 1 [file cells-12-01182-s001.zip › cells-2183337-supplementary/Figure S1.png]

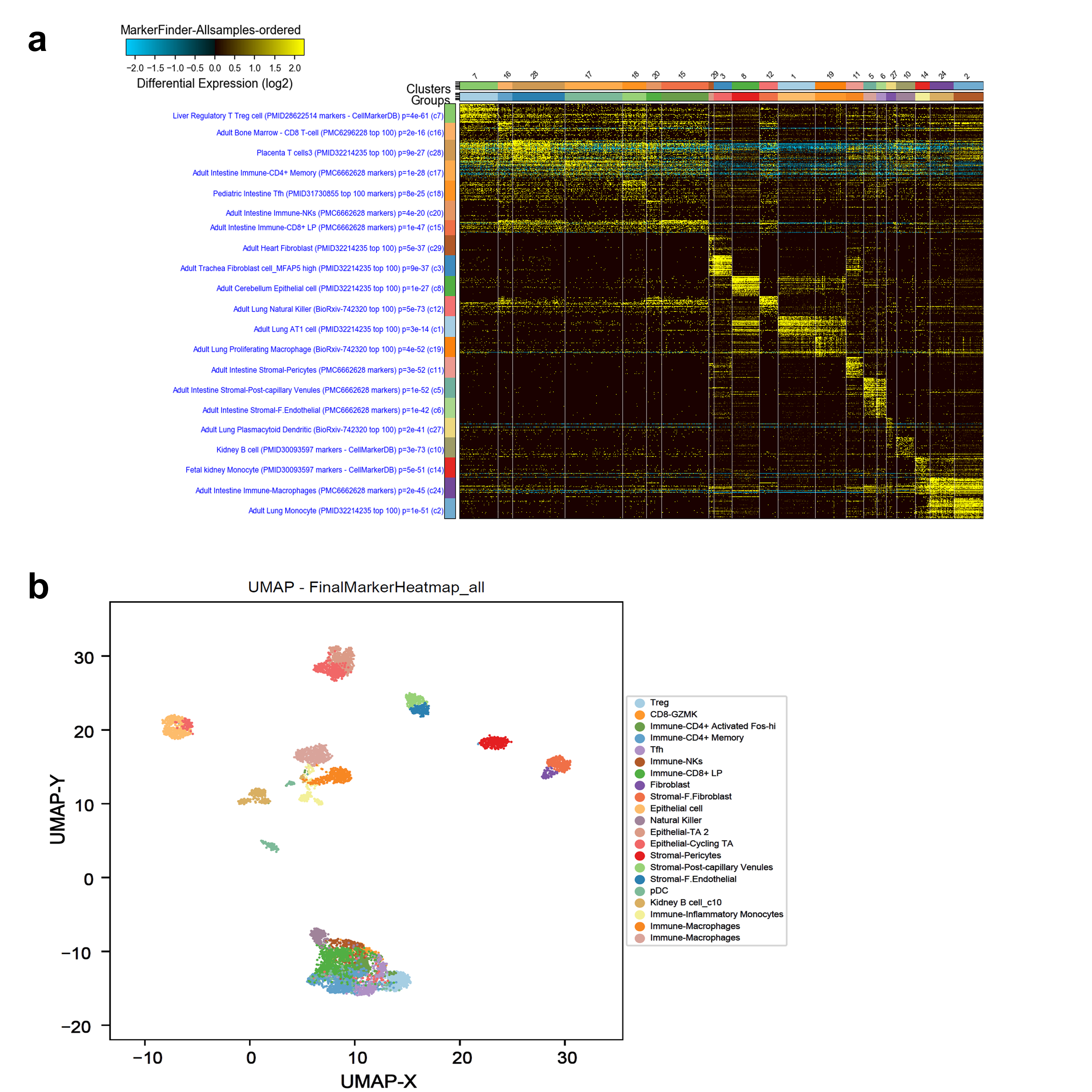

Supplement: Supplementary file 1 [file cells-12-01182-s001.zip › cells-2183337-supplementary/Figure S2.png]

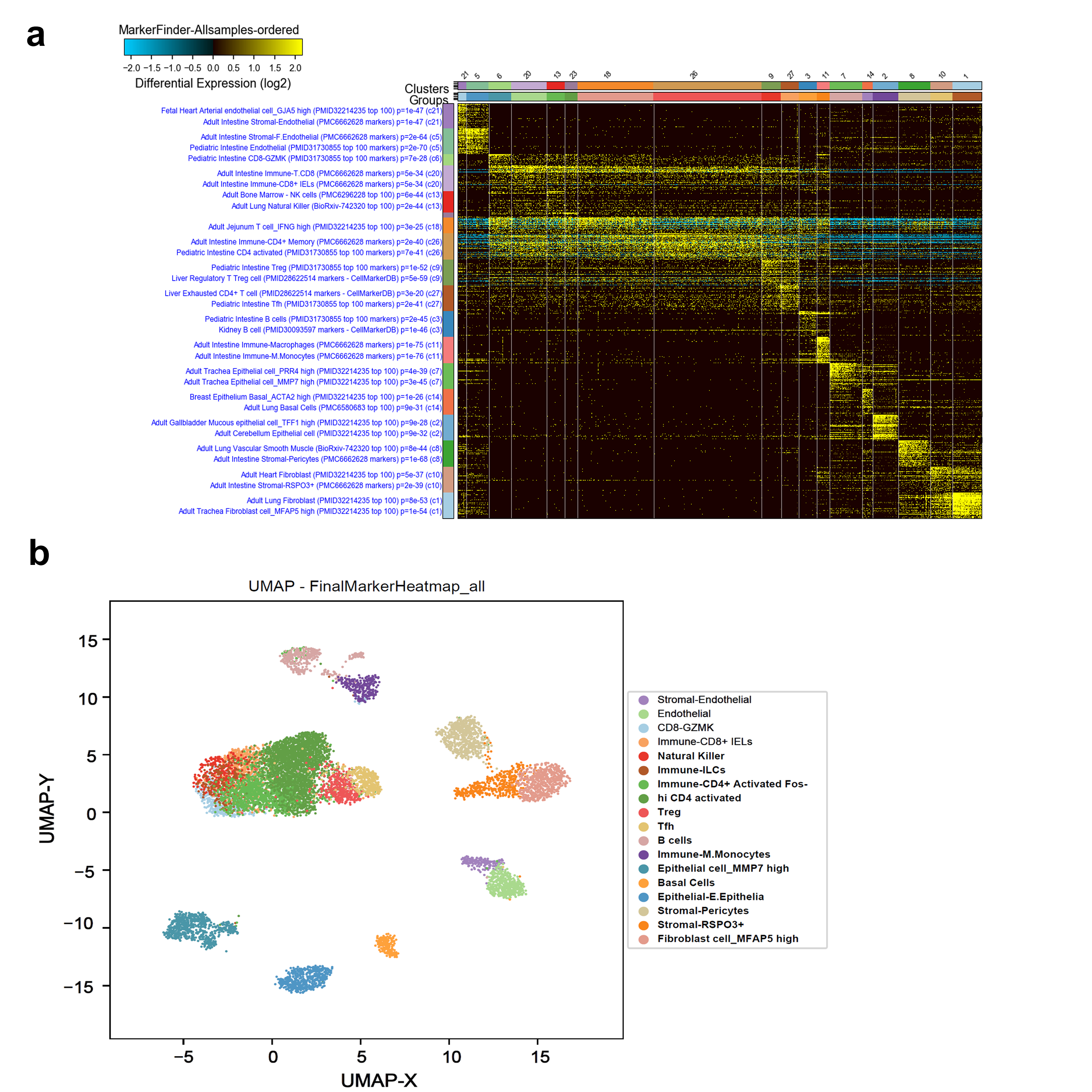

Supplement: Supplementary file 1 [file cells-12-01182-s001.zip › cells-2183337-supplementary/Figure S3.png]

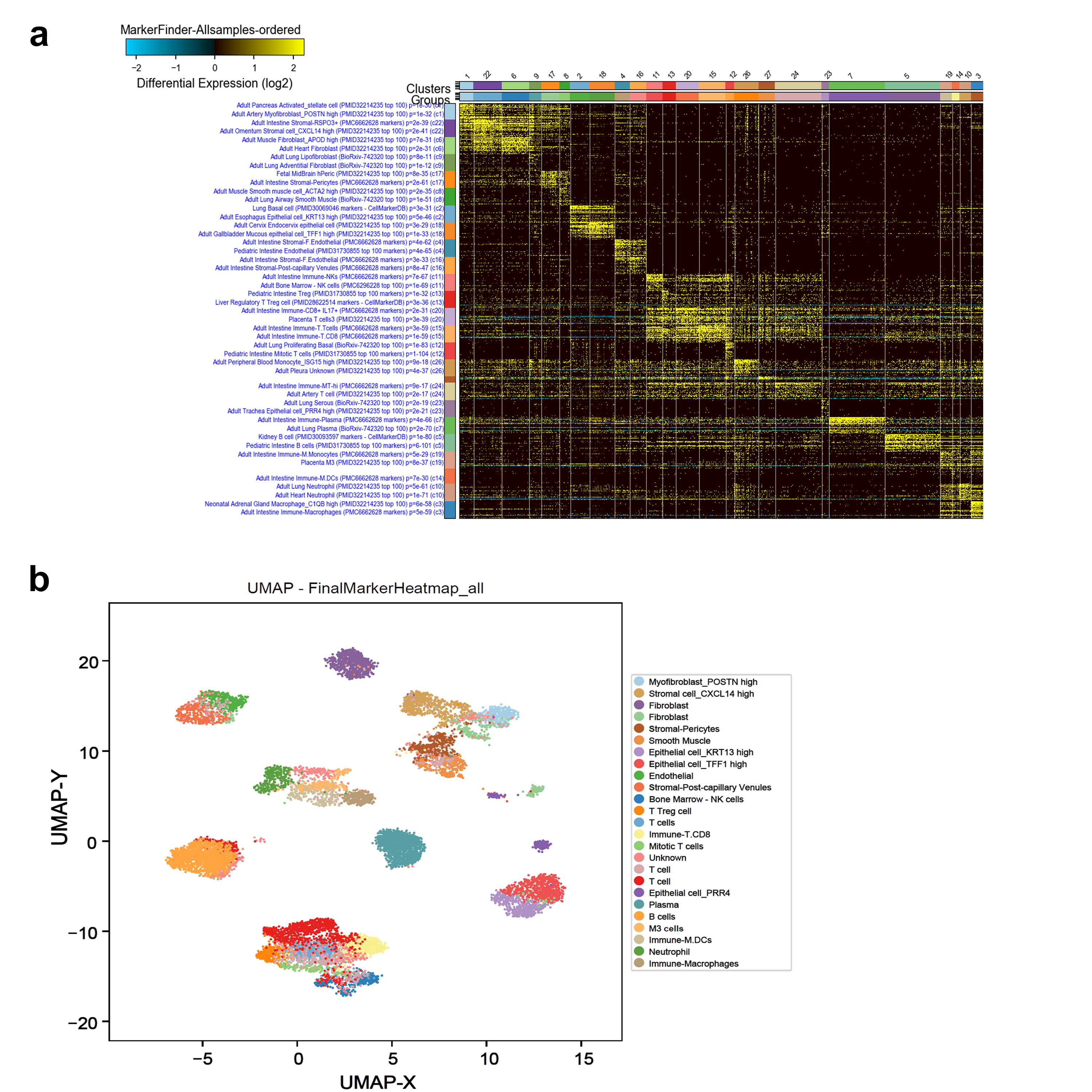

Supplement: Supplementary file 1 [file cells-12-01182-s001.zip › cells-2183337-supplementary/Figure S4.png]

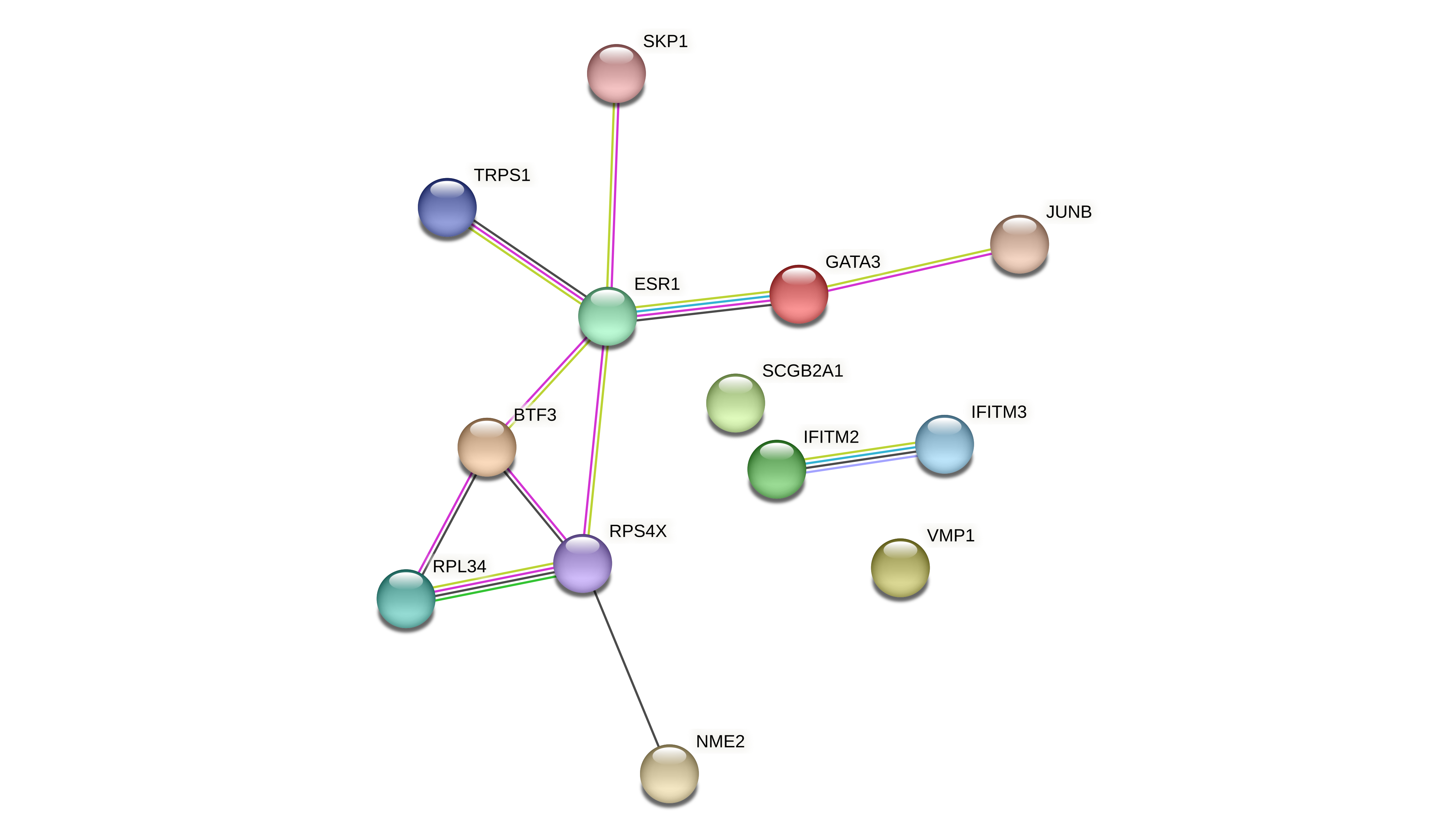

Supplement: Supplementary file 1 [file cells-12-01182-s001.zip › cells-2183337-supplementary/Figure S5.png]

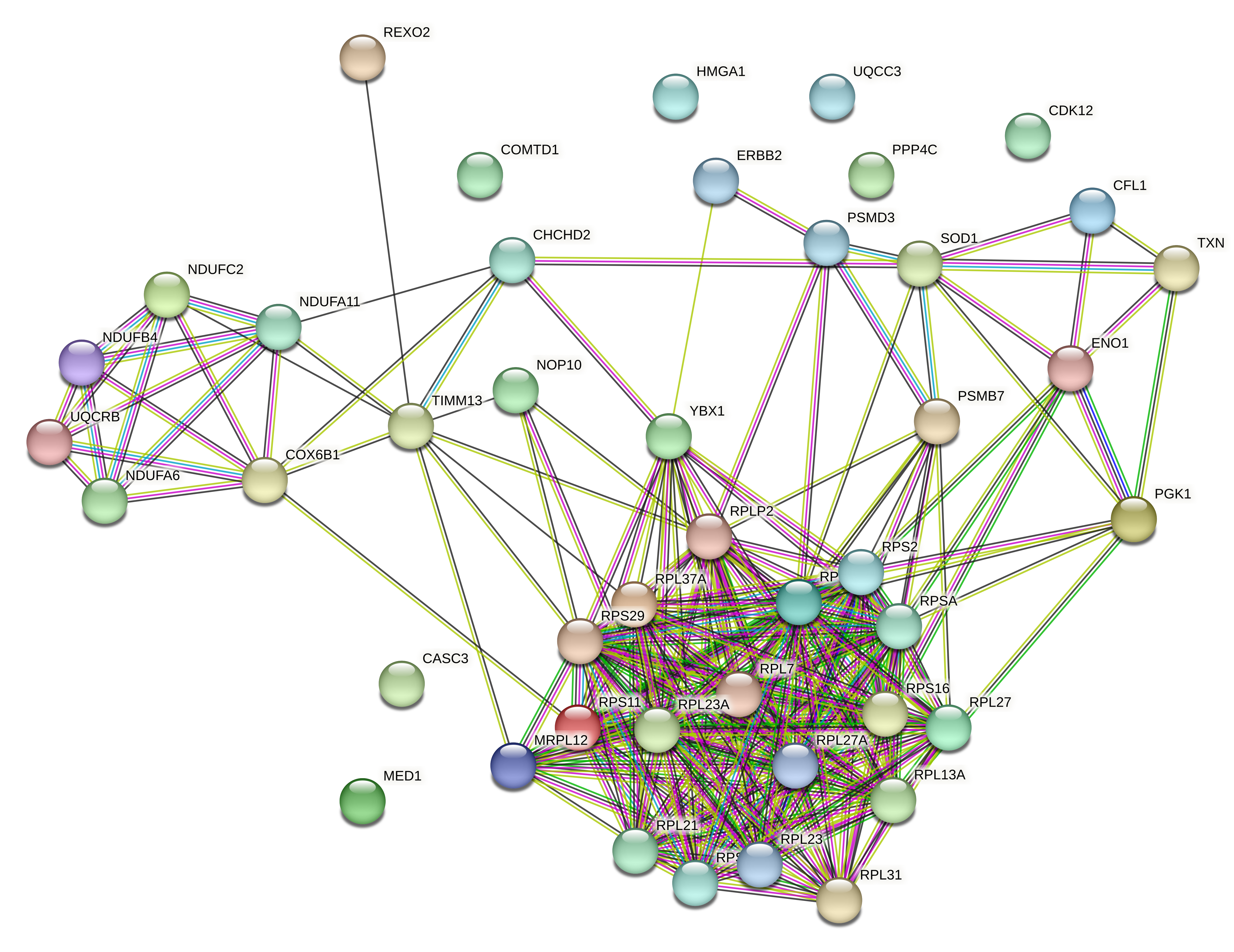

Supplement: Supplementary file 1 [file cells-12-01182-s001.zip › cells-2183337-supplementary/Figure S6.png]

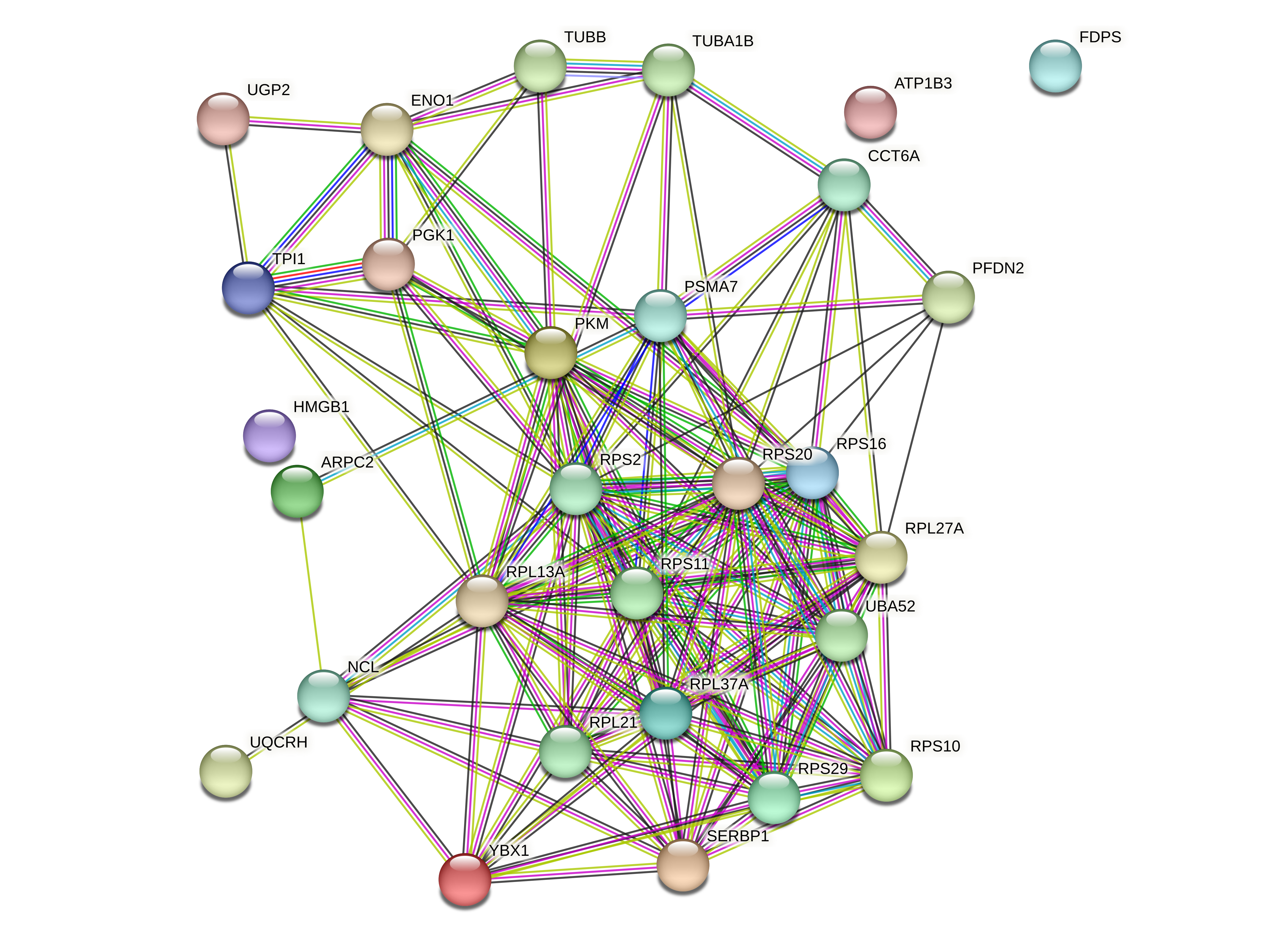

Supplement: Supplementary file 1 [file cells-12-01182-s001.zip › cells-2183337-supplementary/Figure S7.png]

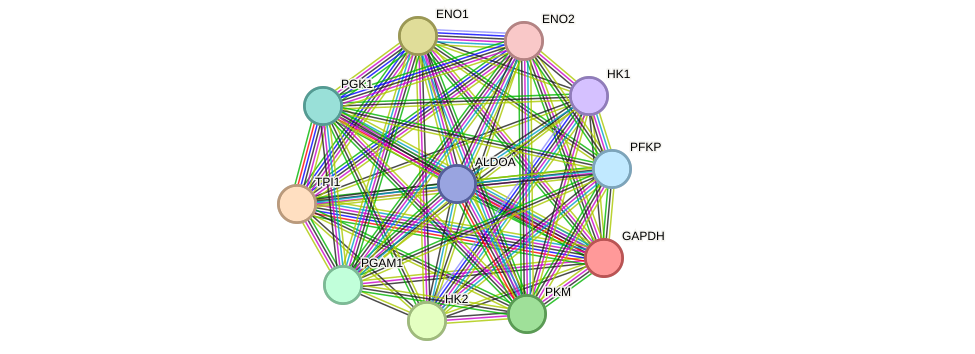

Supplement: Supplementary file 1 [file cells-12-01182-s001.zip › cells-2183337-supplementary/Figure S8.png]

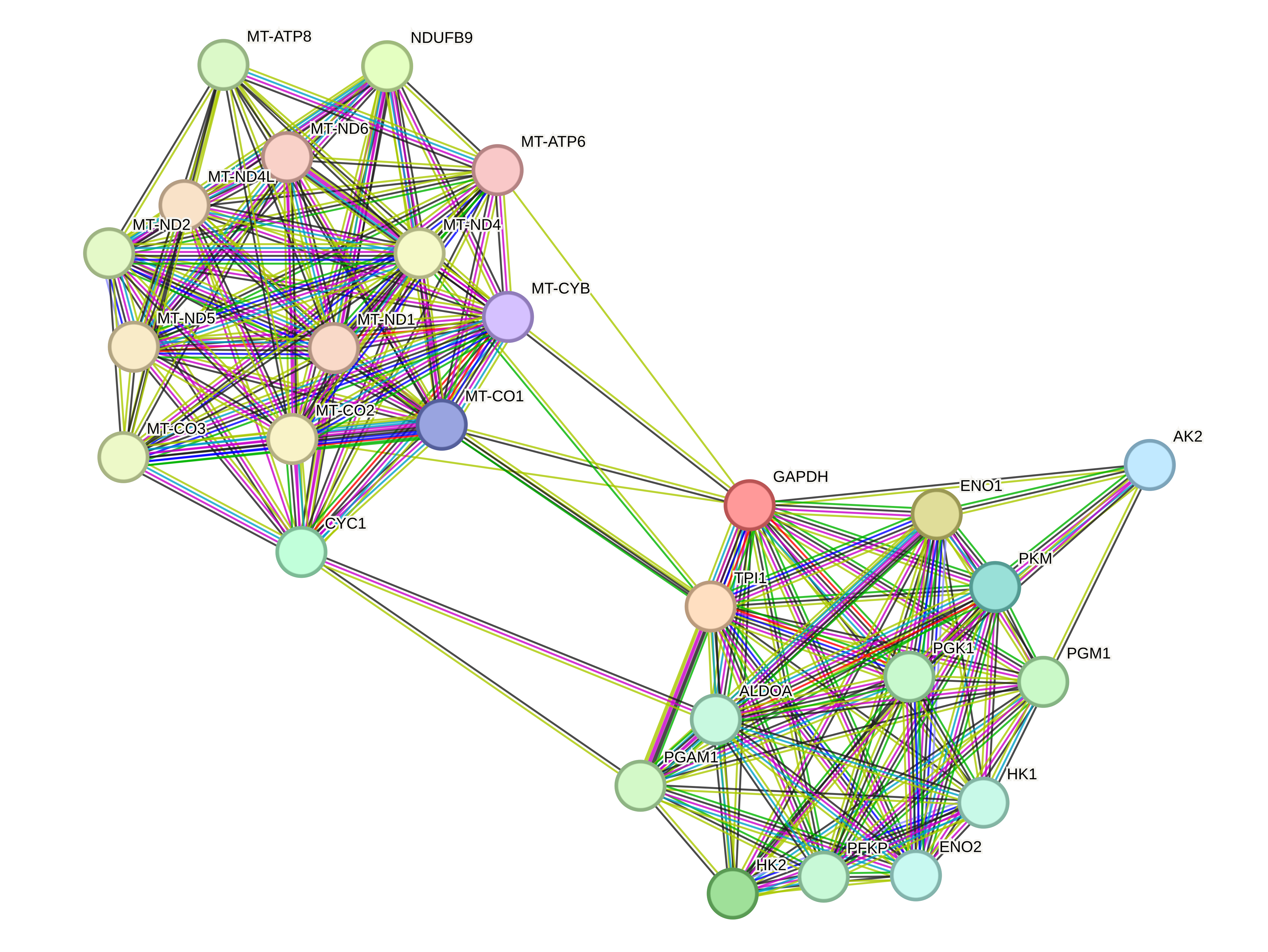

Supplement: Supplementary file 1 [file cells-12-01182-s001.zip › cells-2183337-supplementary/Figure S9.png]
